# Assessment of the Bioaccessibility of Carotenoids in Goji Berry (*Lycium barbarum* L.) in Three Forms: In Vitro Digestion Model and Metabolomics Approach

**DOI:** 10.3390/foods11223731

**Published:** 2022-11-20

**Authors:** Ziying Hu, Yanan Ma, Jun Liu, Yijun Fan, Anran Zheng, Pengyan Gao, Liang Wang, Dunhua Liu

**Affiliations:** 1 School of Food & Wine, Ningxia University, Yinchuan 750021, China; 2 National Key Laboratory for Market Supervision of Quality and Safety of Goji Berry & Wine, Yinchuan 750021, China; 3 School of Agriculture, Ningxia University, Yinchuan 750021, China; 4 School of Statistics, University of International Business and Economics, Beijing 100029, China

**Keywords:** goji berry, bioaccessibility, carotenoids, metabolomics, zeaxanthin dipalmita

## Abstract

Goji berry (*Lycium barbarum* L., LBL) is a good source of carotenoids, while the bioaccessibility of various types of LBL carotenoids has not been explored. In the study, eight carotenoids, three carotenoid esters and two carotenoid glycosylated derivatives were identified by a non−targeted metabolomics approach. The dried LBL (DRI), LBL in water (WAT), and LBL in “Baijiu” (WIN) were used to recreate the three regularly chosen types of utilization, and the in vitro digestion model showed that the bioaccessibility of the carotenoids increased significantly from the oral to the gastric and intestinal phase (*p* < 0.05). The bioaccessibility of LBL carotenoids was the most elevated for DRI (at 28.2%), followed by WIN and WAT (at 24.9% and 20.3%, respectively). Among the three carotenoids, zeaxanthin dipalmitate showed the highest bioaccessibility (51.8–57.1%), followed by β−carotene (51.1–55.6%) and zeaxanthin (45.2–56.3%). However, the zeaxanthin from DRI exhibited significantly higher bioaccessibility (up to 58.3%) than WAT and WIN in both the gastric and intestinal phases (*p* < 0.05). Results of antioxidant activity tests based on DPPH, FRAP, and ABTS showed that the addition of lipids improved the bioaccessibility of the carotenoids. (*p* < 0.05).

## 1. Introduction

Goji berry (*Lycium barbarum* L., LBL) is a dicotyledonous plant in the Solanaceae family. Its organic products are normally dazzling orange−red in variety because of their high carotenoid content, making it an important natural substance for clinical examination and useful food improvement [1]. However, there are numerous factors that may affect carotenoids to get through the cell walls and used by the body for assimilation and retention. As such, the bioaccessibility of carotenoids is affected by many variables. Due to their long linear chains of conjugated π−electron double bonds, carotenoids are susceptible to isomerization, which may be affected by temperature, oxygen content, light exposure, water activity, pH value, or metal content [2,3]. The type of carotenoids in the food matrix, the condition of the food matrix, cell wall structure and composition, and cellular integrity are additional factors that limit the bioaccessibility of carotenoids [4]. Therefore, it is crucial to choose a suitable consumption method for LBL so that carotenoids can be maximally retained, digested, and absorbed by the body.

Among all known dietary sources, radiant red berries contain high levels of carotenoids and zeaxanthin, and are basically present as dipalmitate [5]. It was reported that the primary carotenoid of LBL was zeaxanthin dipalmitate (ZDP) and LBL can be a decent wellspring of ZDP [6,7]. However, the types and contents of carotenoids reported vary due to influencing factors, such as the type of LBL, limitations, and differences in detection methods [8]. As the fruits develop and mature, carotenoids are gradually accumulated in the colored bodies converted from chloroplasts, and the epidermis of the fruit changes from green to bright red [6]. Metabolomic methods are fundamental apparatuses in the food sector to identify metabolites and perform subjective and quantitative analyses [9,10]. Detection systems, such as LC−MS, HPLC−ESI/APCI−MS, and UPLC−LTQ−MS, with high detection sensitivity and phenomenal detachment execution, are commonly used to detect small molecule metabolites in fruits [11,12]. Among them, ESI and APCI techniques are relatively more suitable soft ionization techniques for the analysis of protonated molecular ions of the most relevant plant secondary metabolites, and numerous studies have demonstrated their superiority for several identification errands [13]. Therefore, a metabolomics approach may be an effective strategy for the identification of carotenoids in LBL.

The active ingredients of the extracts from LBL with various treatments were found to be different, and the phytochemical composition of LBL varied from one production area to another [14]. We thus decided to identify carotenoids in the raw material before studying the relationship between the consumption pattern and carotenoid bioaccessibility of LBL. Presently, few studies have been reported using metabolomic approaches to screen carotenoids in LBL. Carotenoids were identified in various maturation stages of LBL using a high−resolution LC−Q−TOF−MS/MS−based metabolomics approach in the study. In light of the conventional consumption of LBL, dried LBL (DRI), LBL in water (WAT), and LBL in Baijiu (WIN) were selected, followed by an in vitro digestion model to assess the bioaccessibility of carotenoids in the digestive fluid through different consumption patterns of LBL. Furthermore, we also investigated the effect of lipid addition on the antioxidant properties of carotenoids in digest and determine the relationship between carotenoids bioaccessibility and antioxidant properties. Our study may provide new ideas for the high−throughput screening and identification of carotenoids in LBL and help select its consumption method.

## 2. Materials and Methods

### 2.1. Plant Materials and Sample Preparation

Summer fruit (NingQi No. 9) was collected in mid-June, autumn fruit was collected in mid−September, and full−growth fruit was collected in mid−June, July, August, and September. All the fruits were obtained from 50 goji berry trees at the Heilan Mountain Farming and Herding Unit 6 Plantation in Xixia District, Yinchuan. Samples were collected and immediately frozen at −80 °C on the same day. Corn oil was purchased from a local supermarket. DRI: natural air-dried LBL; WAT: 20 g LBL were soaked in 80 °C 100 mL water; WIN: 20 g LBL were soaked in 500 mL 38% of Baijiu (Chinese liquor, Grain−based raw materials, a fermented product that is inoculated with natural microorganisms followed by distillation and storage), for 1 month.

### 2.2. Chemicals and Reagents

The standards included β−carotene (≥97%), lutein (≥97%), zeaxanthin (≥97%) and zeaxanthin dipalmitate (≥97%). Enzymes including α−amylase (852 U/mg) from human saliva, trypsin (8 × usp) and lipase (100–500 U/mg) from porcine pancreas and pepsin (≥250 U/mg) from porcine gastric mucosa and porcine bile extract were purchased from Sigma-Aldrich Chemical Co., Ltd. (St. Louis, MO, USA). DPPH (≥97%), FRAP (≥97%) and ABTS (≥97%) were purchased from Aladdin Chemical Co., Ltd. (Shanghai, China). Anhydrous ethanol (≥99.7%) was purchased from Xuzhou Tianhong Chemical Co., Ltd. (Xuzhou, China). Acetic acid (≥99.7%) was purchased from Shanghai Maclean Co., Ltd. (Shanghai, China) Hydrochloric acid (36−38%), anhydrous methanol (≥99.5%), ferric chloride, ferric sulfate, and potassium persulfate were purchased from Sinopharm Group Co., Ltd. (Hong Kong, China). All the chemicals used are of analytical grade.

### 2.3. Surface Color Measurement

The effect of color change on the surface of treated LBL was evaluated using a colormeter (CR−400, Tokyo, Japan). The instrument was calibrated with a white calibration tile as a background before use, the equatorial part of each LBL was aligned with the lens of the colorimeter, and six samples were measured in each group to minimize experimental error. L* value was used as a brightness indicator, indicating blackness (0) or brightness (100), with the larger value indicating a brighter surface of the measured LBL sample, and the magnitude of the value was generally related to the smoothness of the fruit surface, while a* value indicates redness (+) or greenness (−), and b* value indicates yellowness (+) or blueness (−). Based on the values of L*, a*, and b*, the vividness C* = (a*^2^ + b*^2^)^1/2^ was calculated [15].

### 2.4. Observation of the Epidermis Cells of LBL

The above treated samples were slightly drained and cut longitudinally, put into tert−butanol for 10 min and repeated twice, and then put into a refrigerator at 2–4 °C for pre−cooling. Finally, the samples were put into a vacuum freeze dryer for 10 h and observed with an EVO18 scanning electron microscope (Zeiss, Germany) under light protection conditions.

### 2.5. Analysis of Carotenoid by High Resolution LC−Q−TOF−MS/MS

Grind the fresh goji berry fruits into powder after freeze-drying. Approximately 0.1 g of the sample was put into a 15 mL centrifuge tube. Then, 6 mL of a ternary mixture of hexane/acetone/ethanol (2:1:1, *v*/*v*) containing 0.1% BHT were added to prevent oxidation. The material–liquid ratio was 60:1, and the mixture was ultrasonicated at 45 °C for 30 min before cooled to room temperature. The sample was then centrifuged and the supernatant was collected. The extraction was repeated once, and the supernatant was combined. Then, the combined organic extracts were evaporated to dryness at 45 °C under a stream of nitrogen. Finally, 2 mL of methanol/methyl tert−butyl ether (1:1, *v*/*v*) mixture were added to the sample, solubilized, and passed through a membrane for LC−MS/MS analysis.

The ZORBAX Eclipse XDB with a quadrupole time-of-flight (Q−TOF) mass spectrometer (MS−X500R QTOF, SCIEXAB, Huntington Beach, CA, USA) with an electrospray ionization source (ESI) and an atmospheric pressure ionization source (APCI) was used in this work. Data acquisition was performed in information−dependent acquisition (IDA) mode. Samples were collected on an Agilent Infinity Lab Poroshell 120 EC−C18 (3 mm × 100 mm, 2.7 μm, Agilent Technologies, Waldbronn, Germany).

The LC parameters were as follows: solvent A was methanol/methyl tert−butyl ether/water (85:5:10, *v*/*v*/*v*), solvent B was methanol/MTBE/water (11:85:4, *v*/*v*/*v*), flow rate = 0.4 mL/min; gradient program: free carotenoids: 98% A at 0 min, 93% A at 5 min, 73% A at 15 min, 61% A at 23 min, 43% A at 32 min, 0% A at 33 min, 98% A at 37 MS parameters: ionization mode = ESI^+^, APCI^+^; calibration with locked mass; IDA method with the instrument, where the ion source gases 1 and 2 were both 50 psi, CAD gas was 7 psi with the auxiliary gas of nitrogen; spray voltage of 5500 V; collision energy of 10 V; 02 cone well voltage of 80 V; mass range of *m*/*z* 100−1000 Da; scan time = 0.2 S.

### 2.6. Differential Metabolite Analysis

All raw data were analyzed using Neutral Loss MS Finder software (http://prime.psc.riken.jp/, accessed on 10 November 2021). A non−targeted metabolomics workflow was used to find exact molecular weight, primary and secondary mass spectra and annotate metabolites in METLIN using the HMDB metabolomics network (https://metlin.scripps.edu/, accessed on 23 November 2021) database. The ropls v1.19.8 in R version 4.1.2 (R Core Team 2021) was used for principal component analysis (PCA) and partial least squares discriminant analysis (PLS−DA), and plotted using the ggplot2 package [16]. A permutation test with a round−robin test (200 times) was used to validate the model [17].

### 2.7. In Vitro Digestion Model

Oral phase: 1 g of dried LBL and 20 mL sample (water and LBL mixed, Baijiu and LBL mixed) was chopped to simulate oral chewing. Then, 1 mL of 2.3 nM α−amylase solution, 25 μL of 0.3 M CaCl_2_, and 975 μL of distilled water were added, followed by 1% (*w*/*w*) corn oil. The mixture was then placed in a 37 °C water bath for 15 min. The control group was 1 mL of saline. Then, 10 mL of each digestion solution were used for the next stage of digestion.

Gastric phase: 10 mL of the oral digest were added with pepsin working solution in the same volume as the digest (0.32% pepsin was added to the artificial gastric juice (0.4 g NaCl, 1.4 mL HCl)) 45 min before the experiment to configure the pepsin working solution. The pH value of the mixture was adjusted to 2.5 ± 0.1, followed by incubation in an incubator at 37 °C for 2 h (100 r/min), and 10 mL of each digestion solution were used for the next stage of digestion.

Intestinal phase: 10 mL of gastric digest, plus 0.5 mL of small intestine salt solution (3 M NaCl, 0.25 M CaCl_2_), 1.25 mL of porcine bile salt (54 mg/mL, dissolved in phosphate buffer solution), 1 mL of lipase (24 mg/L dissolved in phosphate buffer) and 1.25 mL of trypsin pancreatin (8 mg/mL dissolved in phosphate buffer) were mixed, and then shaken for 2 h (100 r/min) in an incubator at 37 °C. The experiment was conducted in the dark to avoid carotenoid decomposition and isomerization after stirring.

### 2.8. Quantification of Bioaccessible Carotenoids

#### 2.8.1. High−Performance Liquid Chromatography (HPLC)

Carotenoids of the digest were quantified by high−performance liquid chromatography. A Gemini C18 (250 × 4.6 mm, 5 μm; Phenomenex, CA, USA) column was used for the separation. Chromatographic conditions were: column temperature 30 °C, flow rate 1 mL/min, injection volume 20 μL, wavelength 450 nm; mobile phase A: (methanol/water, 95/5, containing 0.1% 2,6−di−tert−butyl−4−methylphenol) mobile phase B: methyl tert−butyl ether (0.1% 2,6−di−tert−butyl−4−methylphenol); gradient elution: elution program: 0 min, 100% A. A standard curve was prepared by weighing a certain amount of β−carotene, zeaxanthin and zeaxanthin dipalmitate standard and preparing them into a concentration gradient of 100 μg/mL, 50 μg/mL, and 50 μg/mL, respectively. The standard solutions were filtered through a 0.22 μm filter and then the samples were stored at −18 °C for further analyses.

#### 2.8.2. Bioaccessibility Calculation

The bioaccessibility of each carotenoid is expressed as a percentage (%) = (A_1_/A_0_) × 100, where A_1_ indicates the amount of each carotenoid in the digest after digestion, and A_0_ indicates the amount of each carotenoid in the digest before digestion.

### 2.9. Determination of Total Carotenoids in Digestive Fluid

Standard curves (5 concentration levels) were prepared using β−carotene, lutein, zeaxanthin, and the zeaxanthin dipalmitate standard in the concentration range of 1–100 μg/mL. The carotenoid content was calculated by the following equation:

(1) $$\mathrm{Carotenoid}\;\mathrm{content}\;(\mu g/g)=\frac{A\times V\times 10^{4}}{\varepsilon^{1\%}\times m}$$

Therein, A is the absorbance value at 450 nm, V is the total volume of the extract (mL), m is the mass of the sample used for the determination (g), and ε is the molar extinction coefficient of β−carotene in hexane (2560 cm^2^/mol).

### 2.10. Determination of Antioxidant Activity

The antioxidant activities of carotenoids in digest were assessed by measuring the 2, 2−diphenyl−1−picrylhydrazyl (DPPH) radical scavenging capacity, ferric ion reducing antioxidant power (FRAP), and 2, 2′−azino−bis−3−ethylbenzothiazoline−6−sulfonic acid (ABTS) radical scavenging capacity [18,19,20].

#### 2.10.1. DPPH Free Radical Scavenging Capacity Assay

DPPH analysis was performed according to the method described by Brand-Williams et al. [18]. Briefly, 0.004 g of DPPH was dissolved in 100 mL of anhydrous ethanol to make DPPH solution, and then 150 μL was taken and reacted with 50 μL of appropriately diluted digestion solution sample for 30 min protected from light. The absorbance value was measured at 517 nm using a spectrophotometer (UV−1800/1800PC, Shanghai Yarong Biochemical Instrument Factory, Shanghai, China). Ascorbic acid was applied as the standard. The DPPH radical scavenging capacity was calculated by the following equation:

(2) $$\mathrm{Scavenging}\;\mathrm{DPPH}\;\mathrm{free}\;\mathrm{radical}\;\mathrm{percentage}\;(\%)=\frac{1-A_{S}}{A_{0}}\;\times \;100,$$

where A_S_ and A_0_ are the absorbance values of the sample solution and the ethanol solution of DPPH (control).

#### 2.10.2. FRAP Iron Ion Reduction Capacity Measurement

FRAP analysis was performed according to the method of Benzie and Strain [19], with some modifications. Briefly, 0.1 M acetate buffer, pH 3.6, TPTZ (10 mM, dissolved in 40 mM hydrochloric acid) and ferric chloride solution (20 mM) were mixed in the ratio of 10:1:1. Then, 5 μL of sample solution were mixed with 150 μL of FRAP reagent. The reaction was carried out at 37 °C in the dark for 40 min, then the absorbance values were measured at 593 nm. The concentration and absorbance curves were plotted with FeSO_4_ solution at concentrations from 0.1 to 1.6 mM, and the absorbance values were brought into the standard curve to calculate the FRAP values.

#### 2.10.3. ABTS Free Radical Scavenging Capacity Assay

ABTS radical scavenging ability was analyzed according to the method of Re et al. [20], with some modifications. Briefly, 440 μL of potassium persulfate (140 mM) were mixed with 25 mL ABTS (7 mM) and kept away from the light for 12−16 h. The mixture was then diluted with methanol to an absorbance value of 0.7 ± 0.02. Then, 5 μL of the sample were reacted with 120 μL of the above solution for 10 min in the dark, and the absorbance value was measured at 734 nm. Trolox was applied as the standard. The ABTS radical scavenging capacity was calculated by the following equation:

(3) $$\mathrm{Scavenging}\;\mathrm{ABTS}\;\mathrm{free}\;\mathrm{radical}\;\mathrm{percentage}\;(\%)=\frac{1-A_{x}}{A_{0}}\;\times \;100,$$

where A_0_ and A_x_ are the absorbance values of the methanol ABTS solution, and the ABTS mixture with the addition of the sample.

### 2.11. Statistical Analysis

All experiments were conducted at least in triplicate. Results are expressed as mean ± standard deviation (*n* ≥ 3) and were plotted by Origin 2018 (OriginLab Inc., Northampton, MA, USA). Data were analyzed by ANOVA, followed by Tukey’s post−hoc test using SPSS.19.0 software (IBM, Chicago, IL, USA). For each analysis, *p* < 0.05 was considered statistically different.

## 3. Results and Discussion

### 3.1. Screening and Identification of Carotenoids

High quality resolution screening locators can be used to determine ions and fragments’ most probable molecular formula to identify unknown compounds. A high−resolution LC−Q−TOF−MS/MS−based framework combined with ESI^+^ and APCI^+^ ionization modes was used for the non-designated location of natural products, as well as fruits at the full development stage, which is the phase of carotenoid collection in LBL [6]. As shown in Table 1, eight carotenoids, three carotenoid esters, and two carotenoid glycosylated derivatives were identified by screening. It was observed that at *m*/*z* 569 ([M+H]^+^), zeaxanthin and isomers were distinguished in the APCI^+^ mode and showed common fragment ions at *m*/*z* 533 ([M+H−2H_2_O]^+^), 551 ([M+H−H_2_O]^+^), which could be eliminated by toluene from the polyene chain (92 AMU) or water loss generated [21]. By combining chromatographic, UV/Vis and mass spectrometric features with authentic standards, we found that β−carotene exhibited protonated molecules at *m*/*z* 537, and fragmentation collision voltages produced a large number of fragment ions at *m*/*z* 413, 444, 445, 533, and 551, and typical fragments were at *m*/*z* 444 in carotenoids mass spectra, where typical ions were formed by protonated molecules [M+H]^+^ formation [22]. It is noteworthy that we observed in the APCI^+^ mode the spontaneous elimination of water from the protonated molecule by the neoflavoplasm, generating precursor ions at *m*/*z* 583 ([M+H−H_2_O]^+^). In addition, a unique fragment ion was found at *m*/*z* 393 in neoflavoplasm, which may be because it is a double bond belonging to the allyl carbon position and undergoes cleavage [22]. Lutein is more polar and therefore was eluted earlier than carotenoids, followed by carotenoid esters. Eight carotenoids and three carotenoid esters were detected in the APCI^+^ mode, and six carotenoids, two carotenoid esters, and two carotenoid derivatives were detected in the ESI^+^ mode, while 9− or 9−(cis)−zeaxanthin, β−cryptoxanthin, α−carotene, 9− or 9−(cis)−β−carotene, and β−cryptoxanthin monopalmitin were detected only in the APCI^+^ mode. Zeaxanthin glucomannan and 1′hydroxy−γ−carotenoid glucomannan were just detected in ESI^+^ mode. Moreover, neoxanthin, 13− or 13−(cis)−zeaxanthin, all−(trans)−zeaxanthin, all−(trans)−β−carotene, zeaxanthin monopalmitate and zeaxanthin dipalmitate were detected in both modes, probably due to the use of ultrasonic extraction and nitrogen blow drying during extraction of carotenoids to maintain the temperature at 45 °C. Most of the natural carotenoids exist in all−trans structures, but appear in cis during heat treatment. Fratianni et al. [8] found the presence of zeaxanthin, lutein, β−cryptoxanthin, and β−carotene in goji berry extract using a reversed phase HPLC system, but no information on structural isomers was reported. The use of high−resolution mass spectrometry was reported to be a better option than UV−VIS or MS/MS in light of the potential for metabolomic screening of carotenoids [23].

### 3.2. Metabolism Profile of Carotenoids

**Figure 1:**
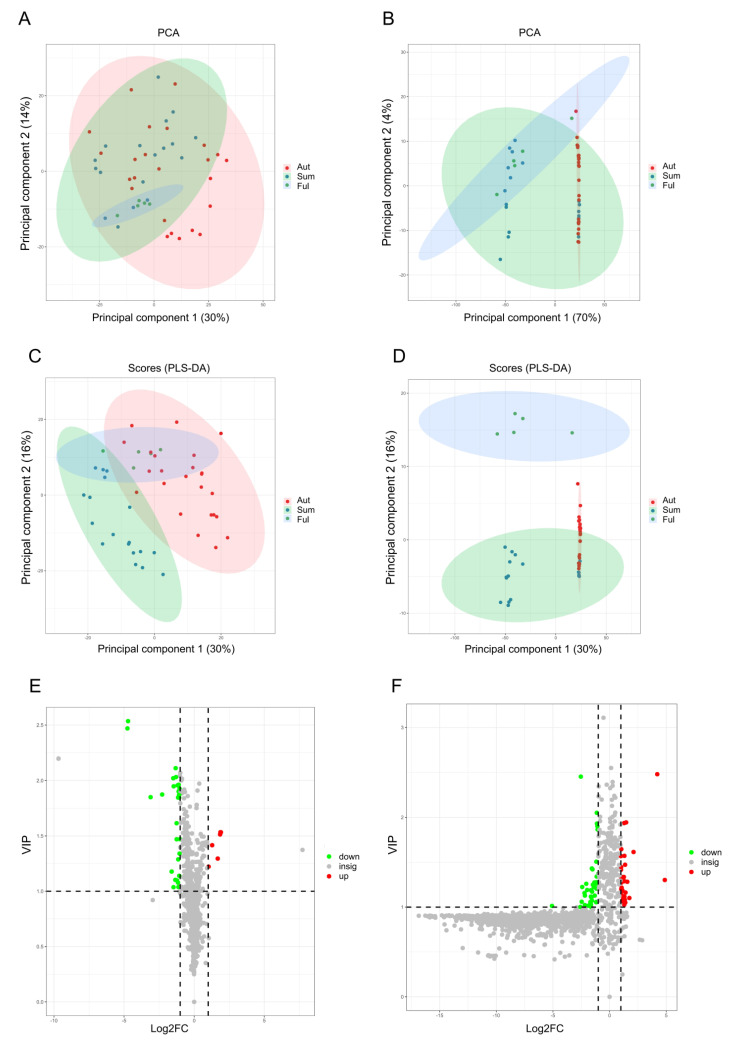
Screening and classification of differential metabolites among summer fruit group (Sum), autumn fruit group (Aut), and full growth fruit group (Ful) in APCI^+^ model and ESI^+^ model. (**A**) PCA score plots of three groups in APCI^+^ model; (**B**) PCA score plots of three groups in ESI^+^ model; (**C**) PLS−DA score plots of three groups in APCI^+^ model; (**D**) PLS−DA score plots of three groups in ESI^+^ model; (**E**) volcano plots for PLS−DA model in APCI^+^ mode; (**F**) volcano plots for the PLS−DA model in ESI^+^ mode. The volcano plot was chosen to identify the differential compounds between the summer fruit group and the autumn fruit group. The horizontal axis is the VIP value and the vertical axis is the fold change (FC). Metabolites with VIP ≥ 1, FC ≥ 2 and FC ≤ 0.5 were selected as the threshold values.

To better demonstrate the validity of our method, a preliminary cluster of differential metabolites among summer fruit group, autumn fruit group, and full growth fruit group was analyzed using PCA (Figure 1A,B), followed by discussion of the specific differentials in different groups using PLS−DA (Figure 1C,D). The root mean square error of cross−validation (RMSECV) and the ratio of standard deviation to the standard error of prediction (RPD) were used to evaluate the accuracy and predictive power of the model [24]. In the APCI^+^ model, the predicted RMSECV value was 0.889% and the RPD value was 3.0; in the ESI^+^ model, the RMSECV value was 0.811%, and the RPD value was 3.3. Typically, the smaller the RMSECV, the higher the accuracy of the model and the better the stability of the model. When the RPD value of the model is greater than 2.5, such treatment can be used for quantitative prediction, and when the RPD value is greater than 3.0, it is suitable for screening and process control, and the higher the RPD value, the better the quantitative results [25]. The differential metabolites were not well distinguished in the APCI^+^ mode in PCA but were completely separated in the ESI^+^ mode. The metabolites monitored by the two models showed different levels of variation and were better discriminated in the ESI^+^ model, indicating significant differences in the carotenoid metabolite profiles between fruit growth periods. The classification results of each group were generally consistent, indicating the reliability of the model we developed in both ion models. The 1384 and 1421 differential compounds were obtained in the APCI^+^ and ESI^+^ mode, respectively, and 89 differential metabolites related to carotenoid metabolism were observed by determining their exact molecular weights. These differential metabolites may be due to significant differences in the contents of soluble solids and soluble sugars in fruits at various ripening stages, while antioxidant substances, such as polysaccharides, phenolics, flavonoids, and vitamin C may synergistically protect carotenoids [14]. This may likewise be strongly connected with soil fertility at different ripening stages. Given the complexity of the carotenoids metabolism profile of LBL, it is of interest to identify them.

### 3.3. Color and Cell Structure Changes of LBL

As shown in Table 2, the values of L*, a*, b*, and C* were higher for DRI. It was reported that the increased contents of zeaxanthin and β−carotene and the decreased level of zeaxanthin dipalmitate in LBL during drying can directly affect the appearance of the fruit [26]. The basic carotenoid structure consists of eight isoprene units with a series of conjugated double bonds that absorb specific wavelengths of visible light, hence giving carotenoids their trademark tone. The principal carotenoid in LBL is ZDP, and therefore exhibited high a* values. In contrast, the low color values in water and Baijiu infused LBL may be because these treatments prompted the disintegration of the fruit epidermal cells, consequently increasing tissue permeability and oxidase activity and finally carotenoid loss.

**Figure 2:**
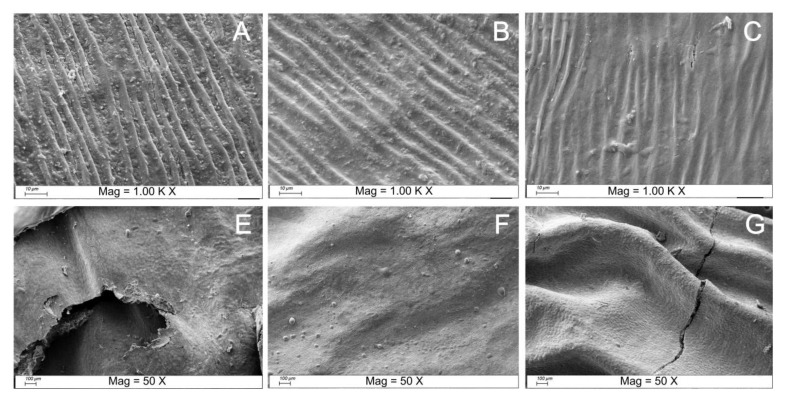
Scanning electron micrographs of the epidermal cells of DRI (**A**,**E**); WAT (**B**,**F**); and WIN (**C**,**G**) at different magnifications.

The cell walls of the fruit provide strong support for the flesh cells, and the intact cell structure is important for maintaining the stability of the intracellular materials [27]. As shown in Figure 2, the DRI lost water inside the flesh cells during natural drying, the cell walls were crumpled, and the epidermal cell structure was severely damaged, thus creating more pores. Many broken cells appeared at the edges of the pores, which may lead to the release of carotenoids. The epidermal cells of WAT were more firmly organized, with the expended cell volumes, and the entire is in striped constriction, with little and more various pores, bringing about better epidermal integrity of the DRI than the WIN. However, this condition may hinder the release of carotenoids. On the other hand, ethanol is less dense than water, with a specific surface tension of 21.97 mN/m and viscosity of 1.074 mPa.s at 20 °C, so the integrity of the epidermal cells was not as good as in WAT, which may contribute to the release of carotenoids. Notwithstanding, the cells were more tightly arranged than DRI, and these peculiarities further uncovered the justification behind the distinction in color values of the three treatments.

### 3.4. Bioaccessibility of Carotenoids

The in vitro digestion method is an effective method to assess the effects of heat treatment, particle size, and oil on carotenoid accessibility. Unlike most other studies using in vitro digestion models, we monitored the bioaccessibility of carotenoids in all three digestion stages for more detailed observation and analysis. The most abundant carotenoid in LBL was ZDP, and the amounts of each carotenoid from LBL used in the study were equivalent to 112.4 μg/100 mL for ZDP, 1.68 μg/100 mL for zeaxanthin, and 29.41 μg/100 mL for β−carotene, in agreement with a previous report [28].

**Figure 3:**
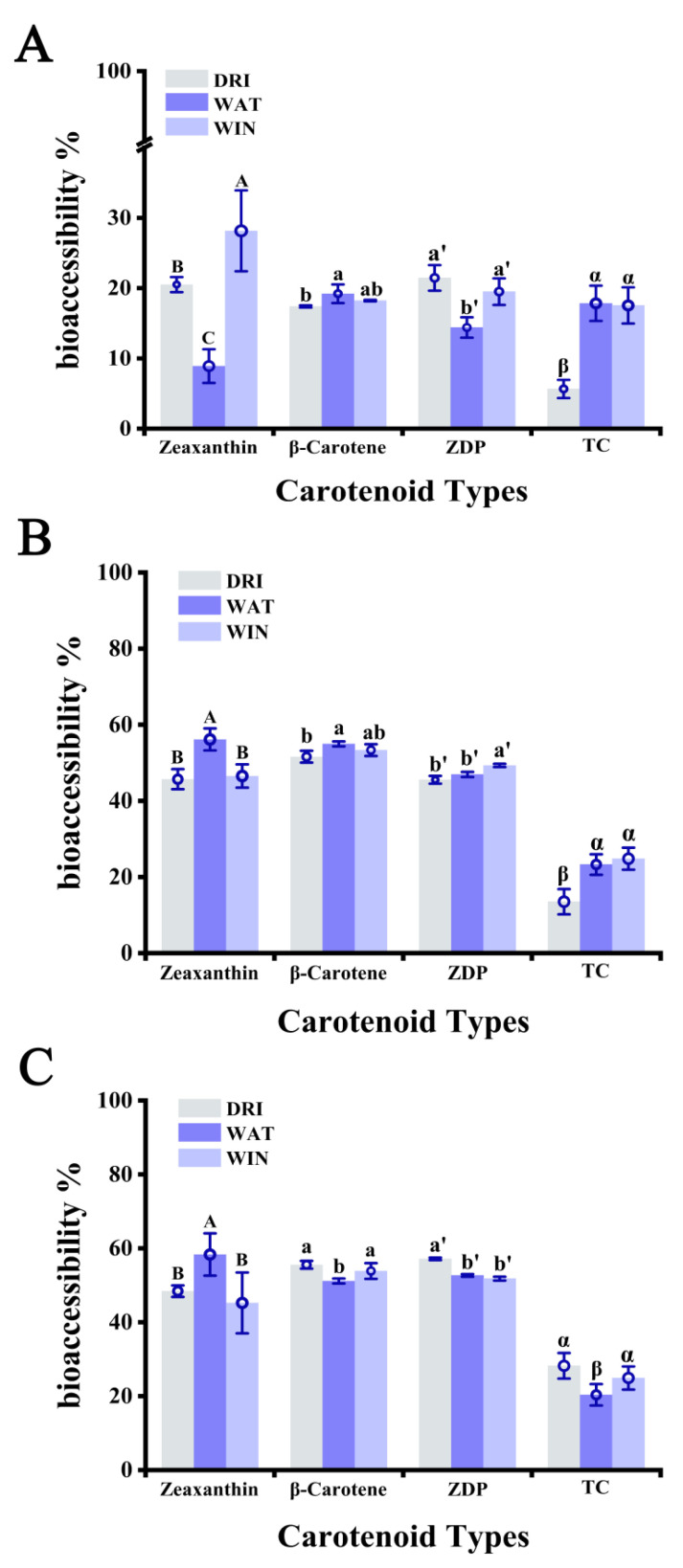
Bioaccessibility of carotenoids in LBL for three kinds of treatments in oral (**A**); gastric (**B**); and intestinal (**C**) digestion stages. DRI represents dried fruits, WAT represents LBL in water, WIN represents LBL in Baijiu, TC represents total carotenoids. The results are expressed as mean ± standard deviation (*n* = 3). Different letters indicate significant differences at *p* < 0.05.

As expected, the values of bioaccessibility in the oral digestion phase were much lower than those in the gastric and intestinal phases (Figure 3A), as there were insufficient digestive enzymes and a hyperosmotic environment to facilitate carotenoids release. In the gastric phase (Figure 3B), the bioaccessibility of zeaxanthin, β−carotene, ZDP and total carotenoids increased by 25.2%, 34.2%, 24.1%, and 7.8%, respectively, for DRI; 47.2%, 35.8%, 32.5%, and 5.4% for WAT; and 18.4%, 35.1%, 29.8%, and 7.3% for WIN. Cell walls and cell membranes exist as physical barriers that retard carotenoids release and digestive enzyme entry, and their integrity is one of the factors that affect carotenoids bioaccessibility in LBL, as the epidermal cells of DRI had relatively low integrity (Figure 2A) and therefore released carotenoids more readily. The bioaccessibility of the carotenoids increased significantly from the oral to the gastric phase, with a progressive increase in bioaccessibility as digestion progressed, suggesting a continuous release and breakdown of carotenoids. In the intestinal phase (Figure 3C), the bioaccessibility of zeaxanthin, β−carotene, ZDP, and total carotenoids of DRI increased by 2.7%, 3.9%, 11.6%, and 14.7%, respectively. ZDP showed the highest bioaccessibility (51.8–57.1%), followed by β−carotene (51.1–55.6%) and zeaxanthin (45.2–56.3%) among the three carotenoids. Nonetheless, a factor that must be considered is the necessity of de−esterification of the monomer or its diester form before the absorption of lutein or zeaxanthin. For DRI, WAT, and WIN, the bioaccessibility rates of total carotenoids were 28.2%, 20.3%, and 24.9% at the final digestion stage, respectively. DRI carotenoids exhibited the highest bioaccessibility. It is worth noting that zeaxanthin from DRI exhibited significantly higher bioaccessibility (up to 58.3%) than WAT and WIN in both the gastric and intestinal phases. One possibility is that the absorption of zeaxanthin requires the presence of dietary fat in the small intestine, which stimulates the release of bile acids, or emulsifiers, from the gallbladder. Bile acids are synthesized in the liver and consist of polar and nonpolar termini that allow the binding of lipophilic and hydrophilic molecules, which may reduce the size of lipid droplets and lead to the formation of mixed lipid micelles [27,29]. The high water content of WAT itself may facilitate the emulsification process and thus the release of zeaxanthin from the cells. In addition, this implies that the absorption of zeaxanthin may require a concomitant higher intake of lipids, which may explain the lower bioaccessibility of WAT and WIN. In addition, zeaxanthin may interact with other carotenoids, and has been reported to reduce the bioavailability of flavonoids [30]. Another possibility is that the fruit cell wall underwent extensive degradation in the gastric phase after exposure to strong acids and digestive enzymes, resulting in small fluctuations in their bioaccessibility in the intestinal phase. It is this fraction of carotenoids that can be digested and absorbed by the body to the greatest extent. Nevertheless, the bioaccessibility of zeaxanthin and ZDP was higher than the bioaccessibility of β−carotene. These results were consistent with a previous study [31]. The bioaccessibility results of WIN for various carotenoids were close to those of WAT (Figure 3C). It was reported that fermentation contributed to carotenoids release and did not affect carotenoid profiles. In addition, alcoholic fermentation was found to decrease the temperature−induced oxidative degradation of carotenoids [32].

In summary, our results suggest that dried goji berries may be the most suitable form of consumption as a carotenoid supplement. Gouado et al. [33] reported that direct consumption had higher carotenoid bioaccessibility among various forms of papaya and mango. Xiang et al. [34] also found that 28 g of LBL taken daily five days a week for three months was effective in carotenoid supplementation, better than a commercially available lutein and zeaxanthin supplement, which is consistent with our results. Although WIN exhibited similar bioaccessibility values to DRI, this consumption option is not suggested due to the health concerns of alcohol. WAT exhibited superior bioaccessibility of zeaxanthin and may be a good choice for zeaxanthin supplementation. It is noteworthy that our results from the in vitro model may be different from the actual values due to the in vivo complexity and individual variability, and thus need to be further validated in an in vivo model.

### 3.5. Antioxidant Activity of Carotenoids in Digestive Fluid

**Figure 4:**
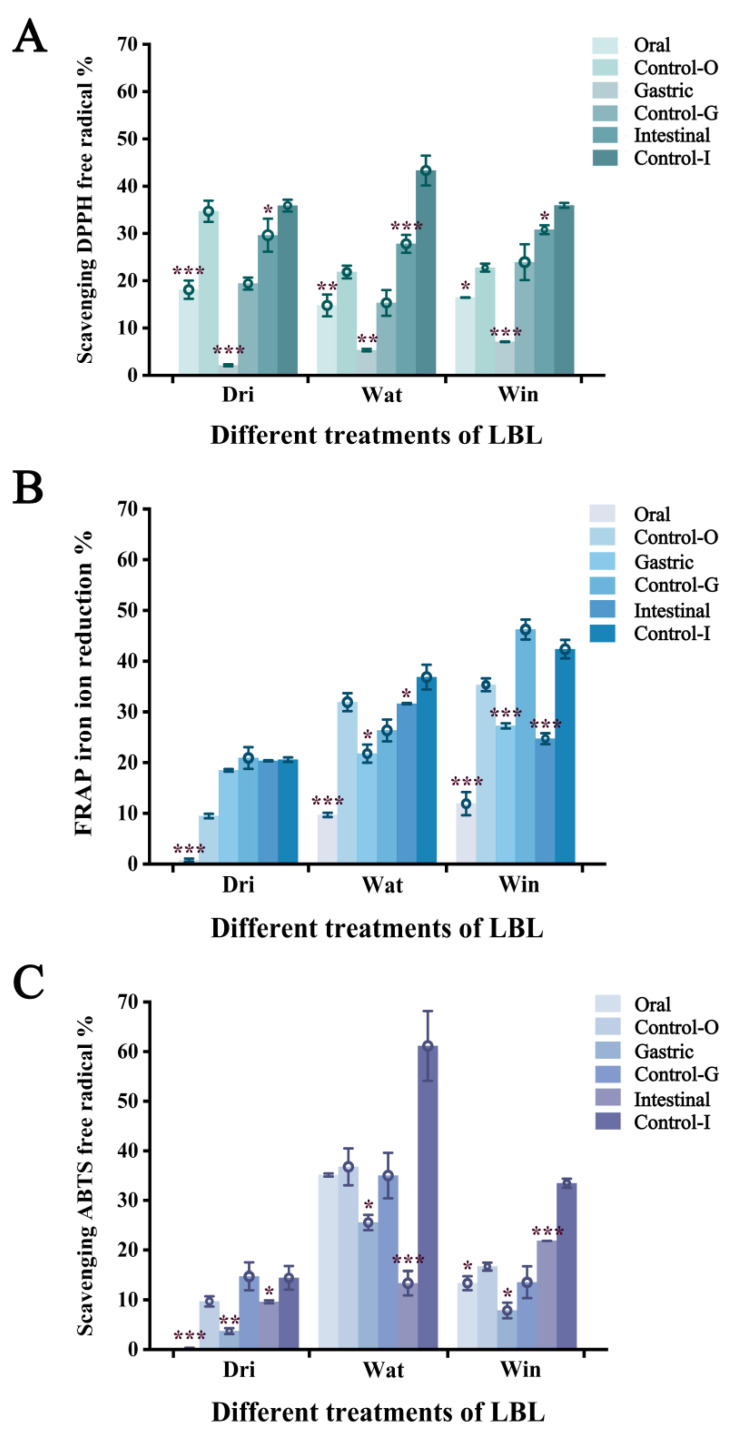
DPPH free radical scavenging capacity (**A**); FRAP iron ion reduction free radical capacity (**B**); and ABTS scavenging capacity (**C**) of LBL in different treatments. The control groups are LBL without corn oil addition in the oral, gastric and intestinal digestive stage (Control−O, Control−G, Control−I). “*” indicates a significant difference between the treatment and control groups (*p* < 0.05), “**” indicates *p* < 0.01, and “***” indicates *p* < 0.001.

Finally, we determined the antioxidant activities of the three carotenoids at the three digestion stages using DPPH, FRAP, and ABTS assays, and the effect of lipid addition. As shown in Figure 4A, 35.9%, 43.3%, and 35.9% of DPPH free radical scavenging capacity were obtained for DRI, WAT, and WIN, respectively. The free radical scavenging capacity decreased significantly (*p* < 0.05) after the addition of lipids. The DPPH free radical scavenging capacity indicates the oxidative free radical scavenging capacity of antioxidants, and that the carotenoid content in the digestive fluid is one of the determinants of the free radical scavenging rate. These results suggest that the added lipids may promote the degradation and isomerization of carotenoids, leading to a decrease in the antioxidant activity of the digest. Similar results were observed for the FRAP ferric ion reduction capacity of DRI, WAT, and WIN (Figure 4B), which decreased from 20.5%, 36.8%, and 42.3% to 20.3%, 31.6%, and 24.7%, respectively, and for the ABTS radical scavenging capacity of DRI, WAT, and WIN (Figure 4C), which decreased from 14.4%, 61.1%, and 33.4% to 9.6%, 13.3%, and 21.8%. Interestingly, DRI exhibited the lowest antioxidant activity in all antioxidant activity tests, further demonstrating the highest bioaccessibility of DRI. Corn oil has longer acyl chains compared to other vegetable oils. It was reported that the micelle core housing carotenoids consisted of monoglycerides and free fatty acids [35], and that the acyl chain length of triglycerides may be positively correlated with the bioaccessibility of carotenoids [36,37]. Similarly, our study has demonstrated the significant effect of lipid addition on the bioaccessibility of carotenoids.

## 4. Conclusions

In this study, eight carotenoids, three carotenoid esters, and two carotenoid glycosylated derivatives were identified with non−targeted metabolomics strategies. In three forms of goji berry consumption, dried goji berry exhibited higher bioaccessibility than water or Baijiu infused goji berry. We found a higher bioaccessibility of water infused goji berry for zeaxanthin and the highest bioaccessibility of zeaxanthin dipalmitate in goji berry carotenoids.

## Figures and Tables

**Table 1:** Mass spectrometric characteristics of carotenoids and their esters in LBL acquired in LC−Q−TOF−MS/MS with APCI^+^ and ESI^+^.

Carotenoids	*m*/*z*	Rt (Min)	Ionic Adducts	Secondary Fragments	Ionization Mode
New yellow mass	601.4	3.43	[M+H]^+^	583/565/491/393	APCI^+^
13− or −13−(cis)−Zeaxanthin	569.4	5.74	[M+H]^+^	469/533/551	APCI^+^/ESI^+^
All−trans−zeaxanthin	569.4	5.77	[M+H]^+^	551/533/543/469	APCI^+^/ESI^+^
9− or 9−(cis)−Zeaxanthin	569.4	5.79	[M+H]^+^	551/533	APCI^+^
β−Cryptophanin	535.4	10.49	[M+H−H_2_O]^+^	461/497/535	APCI^+^
α−Carotene	537.3	13.56	[M+H]^+^	135/331/399	APCI^+^
9− or 9−(cis)−β−carotene	537.4	14.78	[M+H]^+^	445/444/413	APCI^+^
All−trans−β−carotene	537.4	15.62	[M+H]^+^	444/413	APCI^+^/ESI^+^
β−Cryptoxanthin monopalmitate	791.6	45.32	[M+H]^+^	790	APCI^+^
Zeaxanthin monopalmitate	1062	46.93	[M+FA−H]^−^	551	APCI^+^/ESI^+^
Zeaxanthin dipalmitate	1300.1	48.76	[M+FA−H]^−^	533/789	APCI^+^/ESI^+^
Zeaxanthin glucomannan	965.72	8.48	[M+H−H_2_O]^+^		ESI^+^
1′−Hydroxy−γ−carotenoid glucomannan	697.48	4.82	[M−H_2_O−H]^+^	279/78/97	ESI^+^

**Table 2:** Color parameters of LBL with different treatments.

Treatments	Parameters			
	L*	a*	b*	C*
DRI	42.63 ± 1.89 ^a^	46.62 ± 1.01 ^a^	31.72 ± 2.00 ^a^	56.38 ± 1.54 ^a^
WAT	42.80 ± 1.31 ^a^	39.97 ± 3.01 ^b^	30.32 ± 1.74 ^a^	50.16 ± 3.37 ^b^
WIN	39.96 ± 1.14 ^a^	30.28 ± 1.54 ^c^	19.01 ± 2.08 ^b^	35.75 ± 2.19 ^c^

DRI represents dried fruits, WAT represents LBL in water, and WIN represents LBL in “Baijiu”. Values followed by different letters in each line indicated significant differences (*p* < 0.05). The results are expressed as mean ± standard deviation (*n* = 3).

## Data Availability

The data are available from the corresponding author.
